# *Lskipk Lsatpase* double mutants are necessary and sufficient for the compact plant architecture of butterhead lettuce

**DOI:** 10.1093/hr/uhad280

**Published:** 2023-12-28

**Authors:** Sai Xie, Guangbao Luo, Guanghui An, Bincai Wang, Hanhui Kuang, Xin Wang

**Affiliations:** National Key Laboratory for Germplasm Innovation & Utilization of Horticultural Crops; Hubei Hongshan Laboratory; College of Horticulture and Forestry Sciences, Huazhong Agricultural University, 430070 Wuhan, China; National Key Laboratory for Germplasm Innovation & Utilization of Horticultural Crops; Hubei Hongshan Laboratory; College of Horticulture and Forestry Sciences, Huazhong Agricultural University, 430070 Wuhan, China; National Key Laboratory for Germplasm Innovation & Utilization of Horticultural Crops; Hubei Hongshan Laboratory; College of Horticulture and Forestry Sciences, Huazhong Agricultural University, 430070 Wuhan, China; College of Horticulture, Henan Agricultural University, 450002 Zhengzhou, China; North Park, Wuhan Academy of Agricultural Sciences, Wuhu Eco-park, Huangpi District, Wuhan, China; National Key Laboratory for Germplasm Innovation & Utilization of Horticultural Crops; Hubei Hongshan Laboratory; College of Horticulture and Forestry Sciences, Huazhong Agricultural University, 430070 Wuhan, China; National Key Laboratory for Germplasm Innovation & Utilization of Horticultural Crops; Hubei Hongshan Laboratory; College of Horticulture and Forestry Sciences, Huazhong Agricultural University, 430070 Wuhan, China

## Abstract

Lettuce, an important leafy vegetable crop worldwide, has rich variations in plant architecture. Butterhead lettuce, a popular horticultural type, has a unique plant architecture with loose leafy heads. The genetic and molecular mechanisms for such a compact plant architecture remain unclear. In this study we constructed a segregating population through crossing a butterhead cultivar and a stem lettuce cultivar. Genetic analysis identified the *LsKIPK* gene, which encodes a kinase, as the candidate gene controlling butterhead plant architecture. The *Lskipk* gene in the butterhead parent had a nonsense mutation, leading to a partial predicted protein. CRISPR/Cas9 and complementation tests verified its functions in plant architecture. We showed that the loss of function of *LsKIPK* is necessary but not sufficient for the butterhead plant architecture. To identify additional genes required for butterhead lettuce, we crossed a butterhead cultivar and a crisphead cultivar, both with the mutated *Lskipk* gene. Genetic mapping identified a new gene encoding an ATPase contributing to butterhead plant architecture. Knockout and complementation tests showed that loss of function of *LsATPase* is also required for the development of butterhead plant architecture. The *Lskipk Lsatpase* double mutation could reduce leaf size and leaf angle, leading to butterhead plant architecture. Expression and cytology analysis indicated that the loss of function of *LsKIPK* and *LsATPase* contributed to butterhead plant architecture by regulating cell wall development, a regulatory mechanism different from that for crisphead. This study provides new gene resources and theory for the breeding of the crop ideotype.

## Introduction

Plant architecture, including leaf angle, leaf shape, plant height, and the number and angle of branches, is a comprehensive phenotype, which is regulated by diverse genes and environments [1–3]. Traits associated with plant architecture directly affect plant photosynthetic efficiency, nutrient absorption and distribution, and stress resistance, and ultimately affect economic values of crops [1, 3]. Plant architecture is also a sensory trait for ornamental plants and leafy vegetables [4] . The identification of key genes regulating plant architecture and elucidation of their functional mechanism are of great significance for future breeding of crops with the ideotype [1–3].

**Figure 1:**
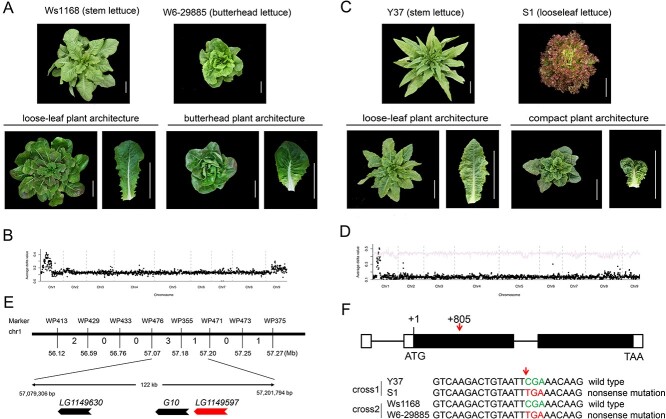
Genetic mapping of the gene controlling butterhead and compact plant architecture. **A** Upper panel, the two parents, a stem lettuce (Ws1168, left) and a butterhead lettuce (W6-29885, right). Lower panel, the two phenotypes of individuals from an *F*_3_ family derived from the Ws1168 × W6-29885 cross. Scale bar = 10 cm. **B** BSR analysis of butterhead plant architecture in the segregating *F*_3_ family in **A**. The *x*-axis represents the nine chromosomes of lettuce. The *y*-axis represents the Δ(SNP index) between two extreme pools. A single locus on chromosome 1 controls plant architecture in the segregating population. **C** Upper panel, the two parents, a stem lettuce (Y37, left) and a loose-leaf lettuce (S1, right). Lower panel, the two phenotypes in an *F*_4_ family derived from the cross Y37 × S1. Scale bar = 10 cm. **D** BSR assay of compact plant architecture in the *F*_4_ family in **C**. **E** Fine mapping of the gene controlling compact plant architecture. Numbers between two neighboring markers refer to the number of recombinants among 4392 individuals in the *F*_4_ family. **F** Gene structure of *LG1149597* (*LsKIPK*), and its sequences in the four parents used in two crosses. The arrow shows the nonsense mutation, converting the codon CGA to TGA.

Lettuce (*Lactuca sativa*) is one of the most important leafy vegetables worldwide. Lettuce is also an ideal crop to be engineered to produce antigen proteins to fight against pathogens such as Ebola virus and COVID-19 [5–7]. Cultivated lettuce was domesticated from its progenitor *Lactuca serriola* in the Caucasus region, and then spread to different parts of the world, where lettuce diverged to several distinct horticultural types, such as butterhead, romaine, crisphead, and stem lettuce [8, 9]. The most popular horticultural types vary among different parts of the world, with stem lettuce as the most popular one in China, crisphead as the most popular one in the USA, and butterhead and romaine as the most popular types in Europe [10]. Crisphead cultivars form ball-shaped crisp leafy heads with leaf bending over the top of the head. Butterhead cultivars, in contrast, produce small spherical heads with pliable leaves and oily texture, with leaf tips on top of the leafy heads. Leafy head is an important agronomic trait for some leafy vegetable crops, and is conducive to mechanized harvesting, transportation, and storage [11]. It has been shown recently that the *LsKN1* gene, upregulated by the insertion of a CACTA-like transposon, contributes to the development of leafy heads in lettuce [12]. The second cloned gene for leafy heads in lettuce is *LsSAW1* [12, 13]. The activated *LsKN1* gene and the mutated *LsSAW1* gene regulate dorsiventrality genes to promote the development of leafy heads [12, 13]. Butterhead lettuce, though with leafy heads, has distinct plant architecture and development processes compared with crisphead. The rate of leaf production and the onset of leafy heads in butterhead lettuce occur later than those in crisphead lettuce [14]. The leaf tips of some butterhead cultivars bend outwards, in striking contrast to the leaf tips of crisphead, which bend inwards. However, it remains unknown whether butterhead and crisphead lettuce share similar genetic or molecular mechanisms for the development of leafy heads.

In this study we examined the genetics underlying the plant architecture of butterhead lettuce. We constructed a segregating population by crossing a stem lettuce cultivar with a butterhead cultivar. An *F*_3_ family derived from this cross was used to clone a gene controlling butterhead plant architecture. The candidate gene was fine-mapped and cloned. Its contribution to butterhead plant architecture was confirmed using a complementation test and CRISPR/Cas9 experiments. Since the first gene is insufficient for the development of butterhead plant architecture, we constructed another segregating population, and genetically cloned the second gene controlling butterhead plant architecture. Different from the mechanism of leafy heads in crisphead, these two novel genes contributed to butterhead plant architecture by regulating the development of the cell wall. Our study provides a new approach and theory for the breeding of the crop ideotype.

## Results

### A locus controlling the plant architecture of butterhead lettuce

To understand the genetics underlying the unique plant architecture of butterhead lettuce, we crossed a butterhead cultivar (W6-29885) with a stem lettuce cultivar (Ws1168) (Fig. 1A). We screened 10 *F*_3_ families, and discovered an *F*_3_ family that showed the segregation of two types of plant architecture, one similar to butterhead and the other similar to loose-leaf lettuce (Fig. 1A). The numbers of plants with the architecture of loose-leaf lettuce (56) and that of butterhead lettuce (22) exhibited a Mendelian ratio of 3:1 (χ^2^ = 0.43 < χ^2^_0.05,1_ = 3.84), suggesting a single gene controlling plant architecture in this *F*_3_ population.

We used bulk segregant analysis (BSA) in combination with RNA-seq (BSA-RNA seq) to dissect the genetics underlying plant architecture in the *F*_3_ population. The plot of Δ(SNP index) showed a single peak on chromosome 1 (Fig. 1B). Using this family, we mapped the gene to the interval of 55 769 129–60 189 439 bp on chromosome 1, harboring 96 predicted genes (Supplementary Data Fig. S1, Supplementary Data Table S1).

**Figure 2:**
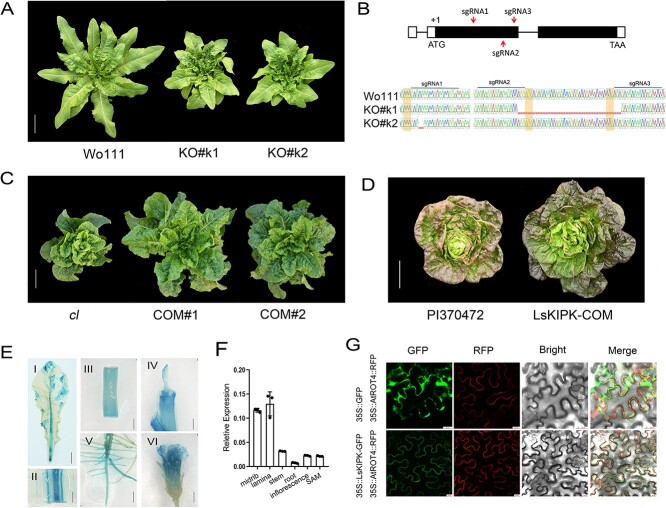
Functional verification of *LsKIPK*. **A** Knockout of *Lskipk* led to compact plant architecture. Scale bar = 10 cm. **B** Upper panel, gene structure of *LsKIPK* and the position of sgRNAs. Black boxes refer to the coding region. Lower panel, Sanger sequencing results showing the modified *LsKIPK* sequences in the two knockout mutants. Dashed lines refer to deletion. Shaded areas refer to the PAM sequences. **C** Complementation test. Transformation of the wild-type *LsKIPK* gene rescued the compact plant architecture. Scale bar = 10 cm. **D** Transformation of the *LsKIPK* gene compromised butterhead plant architecture. Scale bar = 10 cm. **E** GUS staining of leaf (I), midrib (II), stem (III), shoot apical meristem (IV), root (V), and flower (VI) from the *proLsKIPK*::*GUS* transgenic plants. Scale bar =1 cm in (I). Scale bar = 1 mm in (II) to (VI). **F** Expression levels of *LsKIPK* in different tissues (mean ± standard deviation; *n* = 3). **G** Subcellular localization of LsKIPK. Scale bar = 20 μm.

### A locus controlling compact plant architecture overlaps with the locus for butterhead plant architecture

We previously crossed a stem lettuce (Y37) with a loose-leaf lettuce (S1) to construct segregating populations to study the genetics of lettuce (Fig. 1C) [15]. We found that an *F*_4_ family derived from the Y37 × S1 cross showed segregation of two types of plant architecture, one similar to that of loose-leaf lettuce and the other with compact plant architecture (Fig. 1C). Individuals with the loose-leaf plant architecture had flat and long leaves with a large leaf angle, while individuals with the compact plant architecture had twisted and round leaves with a small leaf angle. The numbers of individuals with loose-leaf architecture (104) and compact architecture (31) showed a Mendelian ratio of 3:1 (χ^2^ = 0.30 < χ^2^_0.05,1_ = 3.84). Using the same BSA-RNA seq method as described above, the gene controlling compact plant architecture was mapped to chromosome 1, which overlapped with the gene controlling butterhead plant architecture (Fig. 1B and D, Supplementary Data Fig. S1). We then used 4392 plants from the *F*_4_ family to fine-map the gene controlling compact plant architecture, which was ultimately mapped to an interval of 122 kb between 57 079 306 and 57 201 794 bp on chromosome 1. Only three genes are present in the candidate interval (Fig. 1E, Supplementary Data Table S2). Two of these three genes had no expression difference and their predicted proteins were identical between the two parents. These two genes were therefore excluded as the candidate gene controlling the compact plant architecture. The other gene, *LG1149597*, from plants with compact plant architecture, had a nonsense mutation leading to a predicted shortened protein, while it had an open reading frame and encoded a full-length protein in wild-type plants (Fig. 1F). The *LG1149597* gene encodes a kinase protein, which is orthologous to the KCBP-interacting protein kinase (AtKIPK, AT3G52890) in *Arabidopsis*. We renamed the *LG1149597* gene as *LsKIPK*.

The *LsKIPK* gene is also within the candidate interval for the gene controlling butterhead plant architecture (see above). Interestingly, the parent of stem lettuce Ws1168 has the wild-type *LsKIPK* allele, and the butterhead parent W6-29885 has the mutated *Lskipk* allele. We hypothesized that the same mutated allele, *Lskipk*, led to butterhead plant architecture and compact plant architecture depending on the genetic background.

### Loss of function of *LsKIPK* contributes to compact plant architecture and butterhead plant architecture

To verify that the loss of function of *LsKIPK* contributes to the compact plant architecture, we knocked out the *LsKIPK* gene from a stem lettuce (Wo111) using CRISPR/Cas9 technology. Compared with the Wo111 recipient, the two knockout mutants obtained, KO#k1 and KO#k2, had compact plant architecture (Fig. 2A). PCR sequencing showed that KO#k1 and KO#k2 had a 41- and a 2-bp deletion in *LsKIPK*, respectively, leading to its loss of function (Fig. 2B). A complementation test was used to further verify the function of *LsKIPK* in plant architecture. The 4432-bp full-length wild-type *LsKIPK* was transformed into a compact line (*cl*), which had the mutated *Lskipk* gene. Two positive transformants were obtained, and all of them converted from compact plant architecture to loose-leaf architecture (Fig. 2C). Therefore, both the knockout experiments and the complementation test confirmed that the loss of function of the *LsKIPK* gene led to compact plant architecture.

To test whether the *Lskipk* gene is also responsible for butterhead plant architecture, we transformed the *LsKIPK* complement plasmid into the butterhead cultivar PI370472. One transformant was obtained, which converted the butterhead plant architecture to a loose-leaf architecture (Fig. 2D). The transgenic plant was self-pollinated, and the plant architecture in the *T*_1_ population co-segregated with the T-DNA insertion (Supplementary Data Fig. S2). Therefore, the loss of function of *LsKIPK* also contributes to the butterhead plant architecture.

To investigate the expression pattern of *LsKIPK*, we fused the 1943-bp sequence upstream of the start codon of *LsKIPK* with the *β-glucuronidase* (*GUS*) gene, and transformed it into lettuce. GUS staining of the *proLsKIPK*::*GUS* transgenic lines showed that *LsKIPK* was highly expressed in the midrib and lamina, and moderately expressed in the shoot apical meristem, stem, root, and mature petals (Fig. 2E). RT–qPCR results also showed that *LsKIPK* was highly expressed in the midvein and lamina (Fig. 2F), consistent with the GUS staining results. We then investigated the subcellular localization of the LsKIPK protein. AtROT4-RFP, which is localized in the cell membrane, was used as a positive control [16]. The fluorescence signal suggested that the LsKIPK protein was localized in the cell membrane (Fig. 2G).

### Loss of function of *LsATPase* is required for butterhead plant architecture

**Figure 3:**
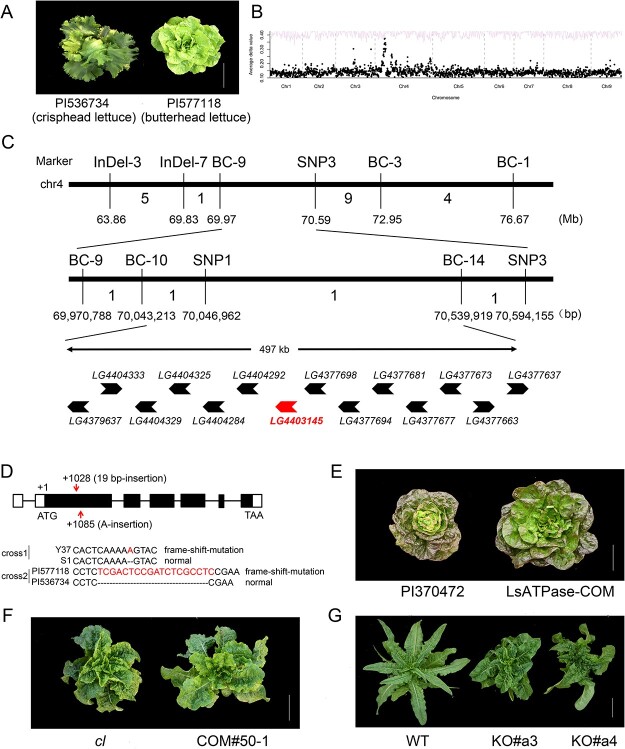
Genetic cloning of the *Lsatpase* gene controlling butterhead plant architecture. **A** Crisphead parent PI536734 and butterhead parent PI577118 used for construction of an *F*_2_ segregating population. Scale bar = 10 cm. **B** BSR analysis of butterhead plant architecture. A significant locus was detected on chromosome 4. **C** Fine mapping of the gene controlling butterhead plant architecture on chromosome 4. **D** Gene structure of *LG4403145* (*LsATPase*), and its sequence polymorphism in the four parents Y37, S1, PI577118, and PI536734 used in the two crosses in this study. **E**, **F** Complementation test in *Lsatpase* mutants. *cl* has the genotype *Lskipk Lsatpase*. Scale bar =10 cm. **G** The *LsATPase* knockout mutant had compact plant architecture. Wild-type (WT) has the genotype *Lskipk LsATPase.* Scale bar =10 cm.

Of the 174 accessions investigated in this study, 70 have the non-functional *Lskipk* allele but did not have a butterhead or compact plant architecture. For example, accessions PI536734, PI229762, and PI178923 had the mutated *Lskipk* allele, but belong to crisphead, romaine, and loose-leaf types, respectively (Supplementary Data Table S3) [9]. Therefore, the loss of function of *Lskipk* is necessary but insufficient for the compact or butterhead plant architecture. To identify a new locus (loci) required for butterhead plant architecture, we crossed a butterhead cultivar (PI577118) with a crisphead cultivar (PI536734), both with the non-functional *Lskipk* allele (Fig. 3A). The *F*_1_ hybrids were self-pollinated to generate an *F*_2_ segregating population. Of the 663 individuals in the *F*_2_ population, 497 had non-butterhead plant architecture and the remaining 166 had a typical butterhead plant architecture, showing a Mendelian ratio of 3:1 (χ^2^ = 5.03 × 10^−4^ < χ^2^_0.05,1_ = 3.84) (Supplementary Data Fig. S3). Using the BSR method as described above, we identified a new locus on chromosome 4 for butterhead plant architecture (Fig. 3B). The causal gene was ultimately mapped to an interval of 497 kb between 70 046 962 and 70 539 919 bp on chromosome 4 (Fig. 3C). There are 14 predicted genes in the candidate interval, including the *LG4403145* gene (Supplementary Data Table S4). *LG4403145* encodes an AAA-ATPase protein containing a DNA-polymerase III γ subunit, and this protein is also localized in the cell membrane (Supplementary Data Fig. S4). The *LG4403145* gene, renamed as *LsATPase*, is wild-type in the crisphead parent, while it is most likely non-functional (*Lsatpase*) in the butterhead parent PI577118 since it has a 19-bp insertion in its coding region (Fig. 3D).

To verify that the loss of function of *LsATPase* is required for the butterhead plant architecture, the 6521-bp wild-type *LsATPase* was transformed into the butterhead cultivar PI370472, and the complementation line LsATPase-COM had a loose-leaf plant architecture, similar to that of the *LsKIPK* complementation line LsKIPK-COM (Figs 2D and3E). To further verify that loss of function of *LsATPase* contributed to compact plant architecture, *LsATPase* was transformed into the *cl* compact line, and, as expected, the transformant COM#50-1 converted from compact plant architecture to loose-leaf architecture (Fig. 3F). We also knocked out the *LsATPase* gene from a loose-leaf plant with the genotype *Lskipk LsATPase*, and the knockout mutants displayed compact plant architecture (Fig. 3G, Supplementary Data Fig. S5). Thus, both the complementation test and the knockout experiments confirmed that the loss of function of *LsATPase* contributed to the butterhead plant architecture and compact plant architecture.

We then revisited the *F*_4_ family derived from the Y37 × S1 cross, which was used to clone the *LsKIPK* gene (see above). We sequenced the *LsKIPK* gene from the two parents, and found a novel non-functional allele of *LsATPase* (*Lsatpase1*), which had an insertion of a single nucleotide, A, in the coding region, while the *LsATPase* gene from parent S1 was wild-type (Fig. 3D). Thus, the Y37 parent had the *LsKIPK Lsatpase1* genotype and the S1 parent had the *Lskipk LsATPase* genotype. The *F*_4_ family, which segregated compact and loose-leaf plant architecture, was homozygous for *Lsatpase1* but heterozygous for *LsKIPK*/*Lskipk*. The results were consistent with our conclusion that the non-functional *Lsatpase* is required for butterhead (compact) plant architecture.

### All butterhead cultivars have the *Lskipk* and *Lsatpase* mutations

**Figure 4:**
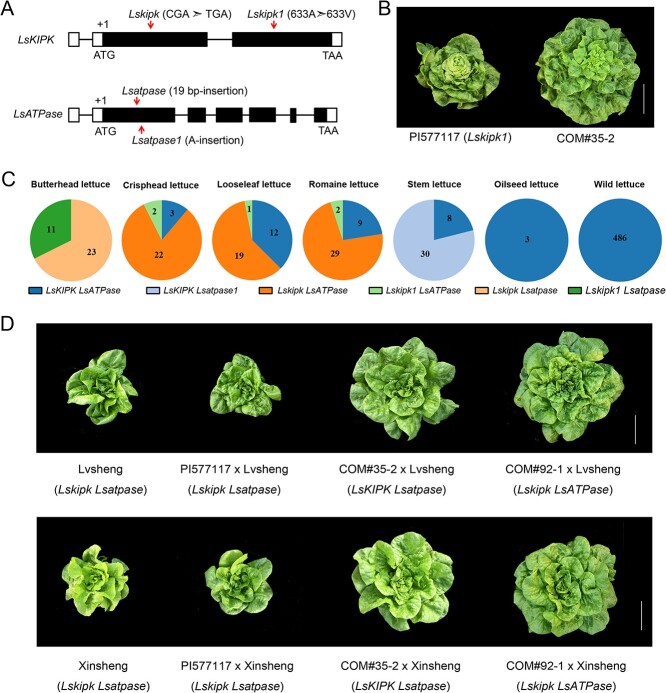
Loss of function of *LsKIPK* and *LsATPase* contributing to butterhead plant architecture. **A** Allele schematic of *LsKIPK* and *LsATPase*. **B** Plant architecture of PI577117 and the complementation line COM#35-2 (*LsKIPK* complementation plant). Scale bar = 10 cm. **C** Distribution of *LsKIPK* and *LsATPase* genotypes in different horticultural types of lettuce. **D** Hybrids between two local butterhead cultivars (Lvsheng and Xinsheng) and PI577117, and hybrids between the two local butterhead cultivars and the complementation lines COM#35-2 (*LsKIPK* complementation plant) and COM#92-1 (*LsATPase* complementation plant). Scale bar = 10 cm.

To further investigate the genotypes of *LsKIPK* and *LsATPase* in different horticultural types of lettuce, we sequenced *LsKIPK* and *LsATPase* from 660 lettuce accessions, including 174 cultivated and 486 wild accessions (Supplementary Data Table S3) [9, 13]. Of the 34 butterhead cultivars screened, 23 had the nonsense mutation (*Lskipk*) (Supplementary Data Table S3). Interestingly, the *LsKIPK* gene from the remaining 11 butterhead cultivars had a distinct allele, which had a point mutation leading to an amino acid change (A633V) in the conserved kinase domain (Fig. 4A, Supplementary Data Table S3). To test whether the allele (named *Lskipk1*) with the A633V mutation contributes to the butterhead plant architecture, the *LsKIPK* complementation vector was transformed into the butterhead cultivar PI577117 (*Lskipk1*). The transgenic plant (COM#35-2) converted from butterhead plant architecture to loose-leaf architecture, showing that *Lskipk1* was also non-functional and contributed to butterhead plant architecture (Fig. 4B). Therefore, all 34 butterhead cultivars included in this study had a non-functional *LsKIPK* (*Lskipk* or *Lskipk1* allele).

We then investigated the genotype of *LsATPase* in lettuce accessions (Supplementary Data Table S3). All 34 butterhead cultivars have the *Lsatpase* allele, consistent with our conclusion that the loss of function *LsATPase* is required for the plant architecture of butterhead lettuce (Fig. 4C, Supplementary Data Table S3). Among the 38 stem lettuces, 30 accessions had the *Lsatpase1* allele (Fig. 4C, Supplementary Data Table S3). Except butterhead and stem lettuce, the *LsATPase* of other horticultural types was wild-type. Therefore, all butterhead accessions were double mutants (*Lskipk Lsatpase* or *Lskipk1 Lsatpase*) (Fig. 4C, Supplementary Data Table S3). In contrast, none of the other horticultural types were double mutants, though 62.5–88.9% were single mutants (Fig. 4C, Supplementary Data Table S3). Notably, all wild lettuces (*L. serriola*) investigated in this study had wild-type *LsKIPK* and *LsATPase*, suggesting that loss of function of *Lskipk* and *Lsatpase* emerged after domestication (Fig. 4C, Supplementary Data Table S3).

**Figure 5:**
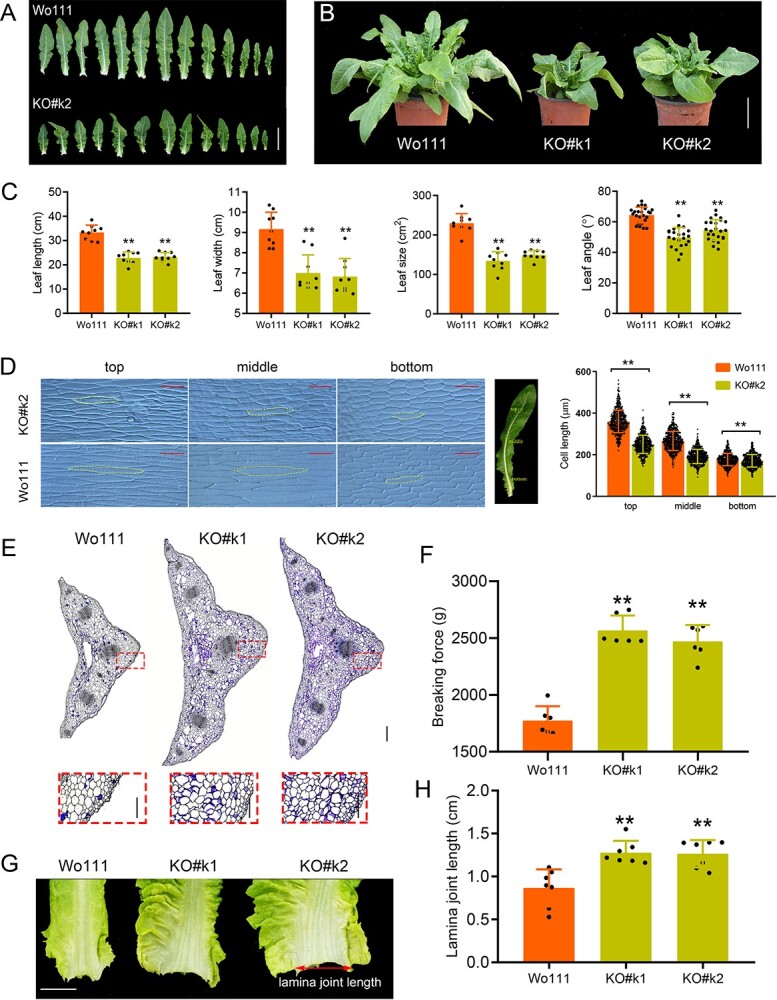
Morphology of *Lskipk Lsatpase* double mutants. **A** Leaves of Wo111 (*LsKIPK Lsatpase*) and knockout mutant KO#k2 (*Lskipk Lsatpase*). Scale bar = 10 cm. **B** Side view of Wo111, KO#k1, and KO#k2. Scale bar = 10 cm. **C** Leaf length (mean ± standard deviation; *n* = 9), leaf width (mean ± standard deviation; *n* = 9), leaf size (mean ± standard deviation; *n* = 9), and leaf angle (mean ± standard deviation; *n* = 24) of Wo111, KO#k1, and KO#k2. **D** Epidermal cell length (mean ± standard deviation; *n* = 646) of leaf midrib between Wo111 and KO#k2. Scale bar = 200 μm. **E** Cross-section of leaf midrib of Wo111, KO#k1, and KO#k2. The rectangular box shows a partial magnification from the large view. Scale bar = 400 μm in large view, and scale bar = 200 μm in box. **F** Minimum force (mean ± standard deviation; *n* = 4) required to break leaf midrib of Wo111and KO#k2. **G**, **H** Morphology (**G**) and length (mean ± standard deviation; *n* = 7) (**H**) of lamina joint. Scale bar = 1 cm. ^*^P < .05, ^**^P < .01. Statistical significance was determined by Student’s *t*-test (**D**, **F**) and ANOVA (**C**, **H**).

To further test whether the loss of function of *LsKIPK LsATPase* is universally required for the development of butterhead lettuce, PI577117 (with butterhead plant architecture) and its complementation lines COM#35-2 and COM#92-1 (with loose-leaf plant architecture; Fig. 4B, Supplementary Data Fig. S6A and B) were crossed with two butterhead cultivars, Lvsheng and Xinsheng, purchased from a local market. The hybrids between PI577117 and the two local butterhead cultivars retained the butterhead plant architecture, while the hybrids between the complementation transformants (COM#35-2 and COM#92-1) and the two butterhead cultivars had loose-leaf plant architecture (Fig. 4D). We concluded that loss of function of *LsKIPK LsATPase* is required for butterhead plant architecture.

### *Lskipk Lsatpase* double mutants had small leaf size and leaf angle

Knockout mutants (*Lskipk Lsatpase*) showed more compact architecture than the control plants (Figs 2A and3G). Leaf length, width, size, and angle of knockout mutants were smaller than in recipient plants (Wo111, *LsKIPK Lsatpase*; wild-type, *Lskipk LsATPase*) (Fig. 5A–C, Supplementary Data Fig. S7A–C). Similarly, the butterhead lettuce was smaller and more compact than its *LsKIPK* and *LsATPase* complementation lines (Supplementary Data Fig. S6A and B). These results revealed that the double mutants (*Lskipk Lsatpase*) but not the single mutants had reduced leaf size. To further identify the effect of *Lskipk Lsatpase* on leaf size, we investigated the epidermal cell morphology of the leaf midrib. Compared with wild-type recipients, the cell length of knockout mutants was significantly shorter (Fig. 5D, Supplementary Data Fig. S7D). Therefore, *Lskipk Lsatpase* reduced leaf size by reducing cell length.

**Figure 6:**
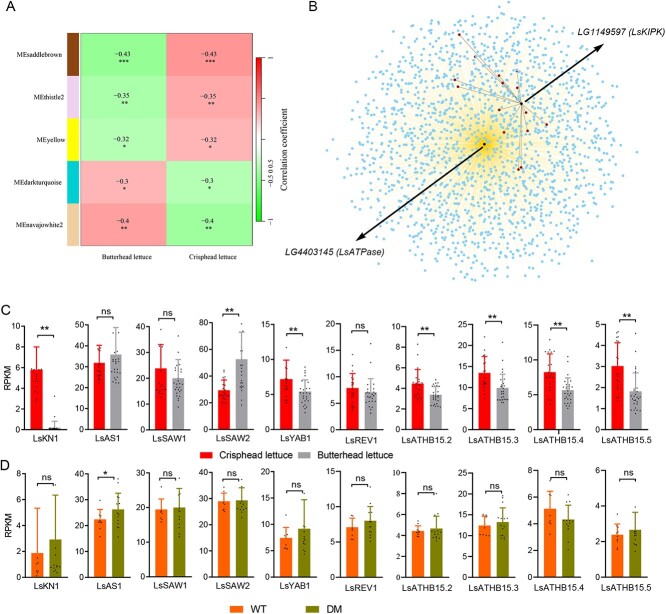
Transcriptome analysis for different genotypes of *LsKIPK* and *LsATPase.***A** Correlation coefficient of modules associated with butterhead and crisphead. Cells contain the correlation value and the asterisk refers to significance. The color gradient scale shows correlations from −1 (green) to 1 (red). **B** Co-expression network of the navajowhite2 module. The two black points refer to *LsKIPK* and *LsATPase*, respectively. Red points refer to co-expression genes shared with *LsKIPK* and *LsATPase*. Blue points refer to co-expression genes with *LsATPase*. **C** Expression pattern of dorsiventrality genes in butterhead and crisphead lettuce. **D** Expression pattern of dorsiventrality genes in wild-type (WT) (wild type, *LsKIPK LsATPase*) and DM (double mutant, *Lskipk Lsatpase*). ^*^P < .05; ^**^P < .01; ^***^P < .001. Student’s t-test was performed in **A**, **C**, and **D**.

To explore the underlying mechanisms of the small leaf angle of knockout mutants, we investigated the leaf midrib and lamina joint. Cross-sections of the leaf midrib showed that the leaf midrib of knockout mutants was wider and thicker than that of the recipients (Fig. 5E, Supplementary Data Fig. S7E). Furthermore, the minimum force required to break the leaf midrib of knockout mutants was significantly higher than that of wild-type recipients (Fig. 5F, Supplementary Data Fig. S7F), and the lamina joint length of knockout mutants was markedly longer than that of recipients (Fig. 6G and H, Supplementary Data Fig. S7G and H). We concluded that *Lskipk Lsatpase* reduced leaf angle by enhancing the mechanical strength of the leaf midrib and lamina joint.

### Comparison between the leafy heads of butterhead and crisphead

Both butterhead lettuce and crisphead lettuce have heading phenotypes. Previous studies have identified several key genes regulating the leafy heads in crisphead lettuce, such as *LsKN1*, *LsSAW1*, *LsSAW2*, *LsAS1*, and *LsYAB1* [12], [13]. To investigate whether butterhead lettuce and crisphead lettuce share similar regulatory mechanisms in leafy heads, we analyzed the transcriptome of a natural population including 21 crisphead lettuce plants and 30 butterhead lettuce plants to construct the co-expression module using weighted gene co-expression network analysis (WGCNA). A total of 58 modules were detected, and five of them were significantly associated with the butterhead or crisphead horticultural type (Supplementary Data Table S5, Fig 6A). Among these modules correlated with the different horticultural types, we found that *LsKIPK* and *LsATPase* co-exist in the navajowhite2 module, which contains 3768 genes, 15 of which were correlated with both *LsKIPK* and *LsATPase* (Fig. 6B). However, *LsKIPK* and *LsATPase* were not correlated with each other. Notably, only *LsYAB1*, a gene related to leafy heads, was in the navajowhite2 module for butterhead. Other dorsiventrality genes, including *LsKN1*, *LsSAW1*, *LsSAW2*, *LsATHB15.5*, and *LsAS1*, were clustered in the lightyellow module, suggesting that this module might be associated with crisphead architecture. We compared the expression of dorsiventrality genes in butterhead and crisphead, and found that *LsKN1* and *LsYAB1* had higher expression, and *LsSAW2* and *LsAS1* had lower expression in crisphead than in butterhead lettuce, which is largely consistent with the results in previous studies (Fig. 6C, Supplementary Data Table S6) [12, 13]. These results implied that dorsiventrality genes critical for crisphead lettuce might not contribute to basic butterhead plant architecture, though they may also cause changes on head shape in some butterhead cultivars.

To further verify the relationship between *LsKIPK LsATPase* and dorsiventrality genes, transcriptomes of 26 progenies derived from the cross of Y37 (*LsKIPK Lsatpase*) and S1 *(Lskipk LsATPase*) were analyzed. Of the 26 progenies, 10 were wild-type (*LsKIPK LsATPase*) and exhibited loose-leaf plant architecture, while the other 16 were double mutants (*Lskipk Lsatpase*) and exhibited compact architecture (Supplementary Data Table S3). Notably, the expression levels of *LsKN1*, *LsSAW1*, *LsSAW2*, and *LsYAB1* were not significantly different between wild-type and double mutants, while the expression of *LsAS1* in double mutants was significantly higher than that in wild-type, suggesting that mutation of *LsKIPK* and *LsATPase* could not affect the expression of most dorsiventrality genes (Fig. 6D, Supplementary Data Table S6). In summary, the major mutated genes controlling butterhead lettuce vary from those controlling crisphead lettuce, though some mutations can be found in both butterhead and crisphead cultivars.

### *LsKIPK* and *LsATPase* function in cell wall and cellulose development

**Figure 7:**
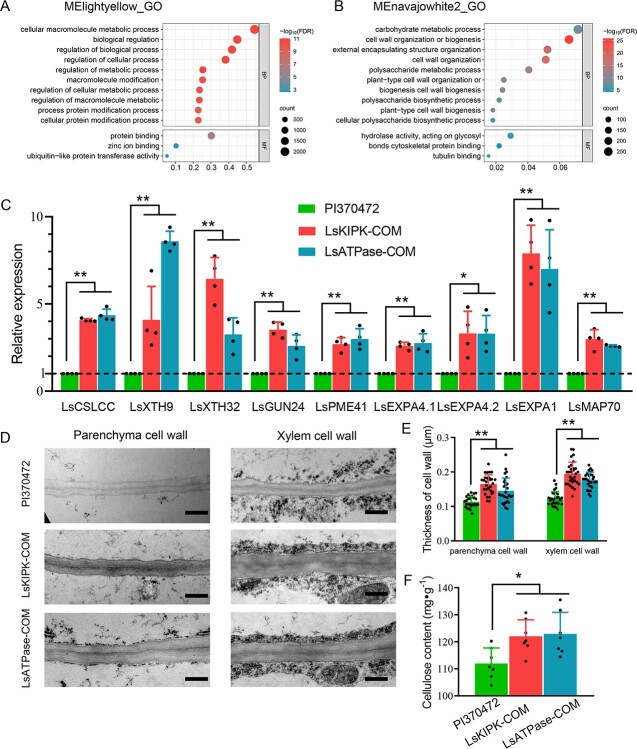
*LsKIPK* and *LsATPase* affect cell wall development. **A** GO enrichment for the lightyellow module, including dorsiventrality genes. **B** GO enrichment for the navajowhite2, module including *LsKIPK* and *LsATPase*. **C** Expression of genes related to cell wall organization or biogenesis (mean ± standard deviation; *n* = 4). **D**, **E** TEM (**D**) and measurement (mean ± standard deviation; *n* = 30) (**E**) of xylem and parenchyma cell wall from PI370472, LsKIPK-COM, and LsATPase-COM. Scale bar = 0.5 μm. **F** Cellulose content (mean ± standard deviation; *n* = 7) of PI370472, LsKIPK-COM, and LsATPase-COM plants. ^*^P < .05; ^**^P < .01. Statistical significance in **C**, **E**, and **F** was determined by one-way ANOVA.

To explore the potential regulatory pathway of *LsKIPK* and *LsATPase*, we analyzed the enrichment of the lightyellow module, including most known dorsiventrality genes, and the navajowhite2 module, including *LsKIPK* and *LsATPase*. Genes in the lightyellow module were mainly enriched in ‘cellular macromolecule metabolic process’ and ‘biological regulation’, while genes in the navajowhite2 module were mainly enriched in ‘cell wall organization or biogenesis’, ‘external encapsulating structure organization’, and ‘polysaccharide metabolic process’ (Fig. 7A and B, Supplementary Data Tables S7 and S8). These findings provided further evidence for the different regulatory mechanisms between butterhead lettuce and crisphead lettuce. The results also implied that the pathways related to cell walls, especially cell wall organization and biogenesis, were crucial for butterhead lettuce.

The navajowhite2 module included *LsCSLCC* (*LG1102483*), *LsXTH9* (*LG3319874*), *LsXTH32* (*LG6528738*), *LsGUN24* (*LG9801327*), *LsPME41* (*LG5476024*), *LsEXPA4.1* (*LG2212177*), *LsEXPA4.2* (*LG9825682*), *LsEXPA1* (*LG8750053*), and *LsMAP70* (*LG4339202*), which were related to cell wall formation (Supplementary Data Table S7). RT–qPCR was performed to quantify their expression in PI370472, LsKIPK-COM, and LsATPase-COM plants. The expression level of these genes in LsKIPK-COM and LsATPase-COM plants was significantly higher than that in PI370472 (Fig. 7C). Collectively, these results revealed that *LsKIPK* and *LsATPase* could affect cell morphology by affecting the expression of genes involved in cell wall development.

We further verified the effects of *LsKIPK* and *LsATPase* on cell structure using transmission electron microscopy (TEM). The cell walls of xylem cells and parenchyma cells in the leaf midrib were significantly thicker in the complementation plants LsKIPK-COM and LsATPase-COM than in PI370472 (Fig. 7D and E). Furthermore, the cellulose content of LsKIPK-COM and LsATPase-COM was significantly higher than that of PI370472 (Fig. 7F). Therefore, *LsKIPK* and *LsATPase* might influence cell morphology and cell wall development so as to contribute to butterhead plant architecture.

## Discussion

### *Lskipk Lsatpase* double mutation is necessary and sufficient for compact plant architecture of butterhead lettuce

Butterhead lettuce is a horticultural type widely used in Europe, accounting for ~80% of lettuce consumption [17]. In this study, we showed that the *Lskipk Lsatpase* double mutation is required for the development of butterhead lettuce. The double mutants have smaller leaf size and leaf angle than the wild-type. The most obvious phenotype of the double mutants is the compact plant architecture, which is the signature of butterhead lettuce. On the background of stem lettuce with apiculate leaf shape, the double mutants also had compact plant architecture, but varied considerably from the architecture of butterhead lettuce (Fig. 2A). In addition to compact architecture, round and soft leaves are also typical characteristics of butterhead lettuce. Therefore, besides the *Lskipk* and *Lsatpase* genes, other genes controlling leaf shape and leaf texture may be also necessary factors for butterhead lettuce.

The *Lskipk Lsatpase* double mutation was found in all butterhead cultivars, consistent with our conclusion that the double mutation is necessary for the development of butterhead cultivars. The double mutation was not found in any non-butterhead lettuce, probably because the double mutation may generate small plants and unappreciated plant architecture and not be selected by breeders. In the *F*_2_ segregating population derived from the cross of butterhead × crisphead, individuals with both the *Lskipk Lsatpase* double mutation and loci controlling leafy heads in crisphead exhibited the butterhead phenotype at the early seedling stage, but developed extremely tight leafy heads at the late development stage (Supplementary Data Fig. S8).

The non-functional alleles *Lskipk* and *Lsatpase* were not found in the wild progenitor of lettuce, and therefore they emerged after domestication. Though *Lskipk Lsatpase* double mutants were found only in butterhead lettuce, the non-functional *Lskipk* allele also had a high frequency in some other leafy lettuce types, such as crisphead. Similarly, the non-functional *Lsatpase* allele had a high frequency in stem lettuce. It remains unknown whether the non-functional alleles of these two genes were artificially selected or maintained at high frequency due to genetic drift.

### Diverse functions of KIPK homologs

The single *Lskipk* mutant of lettuce showed no obvious phenotypic changes compared with the wild-type. Nevertheless, the loss of function of *LsKIPK* caused compact plant architecture on the genetic background of *Lsatpase*. There are two non-functional alleles of the *LsKIPK* gene in nature. The *Lskipk* allele had a nonsense mutation leading to a shortened protein lacking the conserved kinase domain, and allele *Lskipk1* had a non-synonymous mutation (A633V) in the APE motif of the conserved kinase domain. The APE motif stabilizes the activation segment to affect the conformation by phosphorylation [18, 19]. We hypothesized that the A633V amino acid replacement in Lskipk1 may change its kinase conformation, and consequently prevent it from binding to substrates.

KIPK is a member of the AGCVIIIa subfamily of kinases [18]. In *Arabidopsis*, KIPK interacts with KCBP and PERK to negatively regulate root development [20]. Loss of function of a *KIPK* homolog (*dw2*) in sorghum reduces internode length [21]. The diverse phenotypes of *kipk* mutants in different species might be correlated with their distinct expression patterns and subcellular localization. The *DW2* gene in sorghum is highly expressed in developing panicles, peduncles of the inflorescence, internodes, and leaf sheaths. Accordingly, the mutated *dw2* gene reduces cell proliferation at the elongating internodes and changes the morphology of vascular bundles [21, 22]. In contrast, *LsKIPK* is highly expressed in leaf veins of lettuce, and such an expression pattern is consistent with the phenotypes of *Lskipk* mutants, i.e. abnormal leaves (Figs 2E and F and5A and B). The LsKIPK protein in lettuce is localized in the cell membrane (Fig. 2G), while the AtKIPK protein is localized in the nucleus and cytoplasm [23]. Although the localization of Dw2 protein remains unknown, Dw2 is believed to be involved in the maintenance of the endomembrane system and cytoskeleton to regulate endocytosis and alter cell wall polysaccharides, and consequently leads to cell proliferation of the internode [22]. In future, it will be interesting to investigate whether *LsKIPK* has similar functions and whether such functions are associated with the plant architecture of butterhead lettuce.

### Functions of the AAA-ATPase family

Besides the non-functional *Lskipk* gene, the non-functional *Lsatpase* allele was also required for the plant architecture of butterhead lettuce. *LsATPase* is a member of the AAA-ATPase superfamily. This superfamily uses the energy released by ATP hydrolysis to modify their target substrates [24, 25]. AAA-ATPases are macromolecular machines that participate in fundamental cellular processes, including DNA replication, transcription, protein assembly and degredation, and cytoskeleton and membrane remodelling [26]. AAA-ATPase homologs are involved in various plant development processes, such as flowering time, pollen formation, and the development of trichome branches [27–30]. How *LsATPase* functions in the formation of leaf shape and plant architecture remains unclear. More biochemical studies are required in future to understand the molecular mechanism of *LsATPase* in the development of butterhead and to elucidate the relationship between *LsATPase* and *LsKIPK*.

### *Lskipk Lsatpase* controls plant architecture of lettuce by regulating cell wall development

We reported that the *Lskipk Lsatpase* double mutation contributed to compact plant architecture by decreasing leaf size and leaf angle. Moreover, *Lskipk Lsatpase* double mutants could downregulate gene expression relate to cell wall organization and biogenesis and reduce cell wall thickness. Previous studies showed that a thicker secondary cell wall of the leaf lamina joint and leaf midrib could promote tissue mechanical strength and reduce leaf angle, which is the opposite of our results [3]. These reported genes affect leaf angle but not the morphology of leaves. However, the *Lskipk Lsatpase* double mutation affected seveal leaf morphologies, including leaf length and width. Therefore, we hypothesized that the *Lskipk Lsatpase* double mutation could affect leaf angle through multiple pathways, such as cell polarity, cell shape, and the mechanical strength of the leaf midrib.

### Compact architecture lettuce is the ideotype for production

The plant ideotype, including appropriate leaf angle, leaf morphology, and semi-dwarf plant height, reflects the preference of modern breeding. In this study we found that the *Lskipk Lsatpase* double mutation in butterhead lettuce led to a beautiful morphology, improving sensory traits, and to a compact architecture facilitating harvesting and extending shelf life. Our results provided a novel regulatory mechanism for the crop’s ideotype, which could be applied to improvement and germplasm innovation for other crops.

## Materials and methods

### Plant materials and growth conditions

Accessions with names prefixed with CGN were ordered from the Centre for Genetic Resources, the Netherlands (CGN) (http://www.wageningenur.nl/), and accessions with names prefixed with PI or W6 were ordered from the USDA Germplasm Resources Information Network (GRIN) (http://www.ars-grin.gov/). Other accessions were purchased from a local market or collected and maintained by the laboratory. All materials were planted in the open field of Huazhong Agriculture University, Wuhan, China, unless otherwise described.

Several segregating populations were used in this study. Stem lettuce cultivar Ws1168 was crossed with butterhead lettuce W6-29885, and the *F*_1_ hybrids were self-pollinated to generate an *F*_2_ population to study the genetics of butterhead plant architecture. An *F*_3_ family derived from the Ws1168 × W6-29885 cross was used for the genetic analysis of butterhead plant architecture. Segregating populations were previously generated from the cross Y37 × S1 [15]. An *F*_4_ family derived from cross Y37 × S1 was used for the genetic analysis of compact plant architecture. Butterhead cultivar PI577118 was crossed with crisphead cultivar PI536734 for the genetic analysis of butterhead plant architecture.

### BSA-RNA seq

BSR-seq was performed as previously described [15]. An *F*_3_ family, which was derived from a cross between butterhead lettuce W6-29885 and stem lettuce Ws1168, was chosen to identify the locus controlling butterhead plant architecture. From this family, we chose 20 plants with butterhead plant architecture and 20 plants with loose-leaf architecture to construct a butterhead tissue pool and a loose-leaf tissue pool, respectively. Total RNA of the tissue pools was extracted using TransZol Up (Transgen, Beijing, China). Paired-end sequencing of the RNA was carried out on an Illumina HiSeq 2500 instrument in Personalbio, Shanghai, China. About 5 G clean reads of these two pools were obtained and aligned to the lettuce genome assembly v8 [31]. SNPs between the two pools were identified using SAMtools [32]. The frequency of each SNP for the two pools was calculated. The difference in allele frequency [Δ(SNP index)], for each SNP locus between the two pools was calculated. An average Δ(SNP index) was calculated using a 3-Mb window length with a 1-Mb window step, and was plotted along the nine chromosomes of lettuce.

An *F*_4_ family from a cross between the loose-leaf cultivar S1 and stem cultivar Y37 was chosen to identify the locus controlling compact plant architecture [15]. Twenty plants with compact architecture were randomly chosen to construct the compact pool, and 20 plants with loose architecture were randomly chosen to construct the loose pool. The BSA analysis of compact architecture was performed as described above. Genetic mapping primer were listed in Supplementary Data Table S9. 

The *F*_2_ population from a cross between butterhead cultivar PI577118 and crisphead cultivar PI536734 was chosen to identify the locus controlling butterhead plant architecture. Thirty-two plants with butterhead plant architecture were randomly chosen to construct the butterhead pool, and 34 plants with non-butterhead plant architecture were randomly chosen to construct the non-butterhead pool. The BSA analysis of compact architecture was performed as described above. Genetic mapping primer were listed in Supplementary Data Table S9. 

### Constructing vectors and transformation

For the complementation vector, 4432-bp full-length *LsKIPK* and 6521-bp full-length *LsATPase* were amplified using Phanta Max Super-Fidelity DNA Polymerase (Vazyme, Nanjing, China), and pK7LIC1 vector was digested by SmaI (FD0664, Thermo Scientific). Both purified PCR products and the linearized plasmid were recombined using a ClonExpress II One Step Cloning Kit (Vazyme, Nanjing, China). Primer were listed in Supplementary Data Table S10. The recombined plasmids were transformed into *Agrobacterium tumefaciens* strain GV3101 using the freeze–thaw method.

For gene editing, specific sgRNAs for *LsKIPK* and *LsATPase* were designed by CRISPR-P v2.0 (http://crispr.hzau.edu.cn/cgi-bin/CRISPR2/CRISPR) [33]. The purified PCR products containing sgRNA were cloned into pKSE401 vector [34]. Primer were listed in Supplementary Data Table S10. Subsequently, this vector was transformed into *A. tumefaciens* strain GV3101 using the freeze–thaw method.

For plant transformation, firstly the seeds were treated using 50% bleach for 15 min, and were then washed with sterile water. The sterilized seeds were germinated on ½-strength Murashige and Skoog (MS) solid medium (pH 5.6–5.7) at 22°C in a growth chamber. Six days later, cotyledons were harvested and soaked in a suspension of *A. tumefaciens* suspended in full-strength MS liquid medium (pH 5.6–5.7) for 3 min. The treated cotyledons were transferred into full-strength MS solid medium without antibiotics for 36 h. Subsequently, the cotyledons were transferred into full-strength MS solid medium (pH 5.6–5.7) with 0.05 mg/l NAA (naphthaleneacetic acid), 0.5 mg/l KT (kinetin), 60 mg/l kanamycin, and 300 mg/l Timentin and grown at 22°C in a growth chamber. After ~30 days, plantlets were regenerated and transferred into ½-strength MS solid medium (pH 5.6–5.7) with 300 mg/l Timentin. Finally, transgenic plants were transplanted into soil. Wo111 (*LsKIPK Lsatpase*) and wild-type (*Lskipk LsATPase*) plants were used for the knockout assay. The compact line (*cl*), PI370472, and PI577117 were used for complementation tests.

### Subcellular localization

To generate subcellular localization vector, we cloned the coding regions of *LsKIPK* and *LsATPase* using leaf cDNA and Phanta Max Super-Fidelity DNA Polymerase (Vazyme, Nanjing, China), and the purified PCR products were recombined into pK7LIC6 vector using the ClonExpress II One Step Cloning Kit (Vazyme, Nanjing, China). Primer were listed in Supplementary Data Table S10. The recombined plasmids were transformed into *A. tumefaciens* strain GV3101 using the freeze–thaw method. The vector was transformed into *Nicotiana benthamiana* with *A. tumefaciens*, and GFP fluorescence was assayed using a Leica SP8 microscope under 488 nm excitation and 505–545 nm emission.

### Transcriptome, co-expression module analysis, and Gene Ontology analysis

Raw RNA-seq reads were processed using fastp [35] to remove adapter and low-quality sequences. The resulting high-quality cleaned reads were aligned to the ‘Salinas’ lettuce reference genome with gene models using HISAT2 [36]. Following alignment, raw counts for each lettuce gene were normalized to reads per kilobase million (RPKM).

Co-expression network analysis was performed using the WGCNA package in R [37]. A signed hybrid network for normalized expressed genes was constructed using the automatic network construction function blockwiseModules with default settings, except that power = 12 and TOMType = ‘unsigned’. Finally, the network was visualized using Cytoscape v3.6 [38].

For Gene Ontology (GO) enrichment analysis, differentially expressed genes with statistical significance were submitted to AgriGO v2.0 (http://systemsbiology.cpolar.cn/agriGOv2/) [39]. Significant GO enrichment was screened by FDR < .05 and *P* < .05. GO analysis data are listed in Supplementary Data Tables S7 andS8.

### RT–qPCR

A total of 1 μg RNA was used for reverse transcription. cDNA was synthesized using HiScript II Q RT SuperMix for qPCR (+gDNA wiper) (R223, Vazyme, Nanjing, China). RT–qPCR was performed using ChamQ Universal SYBR qPCR Master Mix (Q711, Vazyme, Nanjing, China). Each reaction consists of 2 μl cDNA (10 ng), 0.2 μl primer, 5 μl 2 × ChamQ Universal SYBR qPCR Master Mix, and 2.6 μl ddH_2_O. The reaction was carried out using a two-step method, and the PCR reaction was performed using the QuanStudio™ 6 Flex Real-Time PCR System. The housekeeping gene *UBIQUITIN* (*LG416296*) was used as a control. Primer were listed in Supplementary Data Table S10. Three biological replications and four technical replications were performed for the RT–qPCR. Relative expression levels were calculated using the 2^−ΔΔCt^ (Livak) method with a Bio-Rad protocol.

### Paraffin sections

For leaf tissue sections, leaf tissue was fixed in 50% FAA (50% ethanol, 5% acetic acid, and 3.7% formaldehyde). After 10 h of fixation, samples were dehydrated using a series of ethanol solutions (50, 70, 80, 90, 95, and 100%) for 5 h. The ethanol was replaced using a xylene series (20, 50, 75, 100%) for 5 h. Then, the xylene was replaced using paraffin at 60°C for 16 h. Finally, the tissues were embedded in paraffin wax. Paraffin sections were made using a rotary microtome (Leica), and the slides were stained using toluidine blue. Images were taken using a Leica microscope under bright-field illumination.

### Transmission electron microscopy and determination of maximum shearing force

TEM was performed to investigate the cell morphology of the leaf midrib of PI370472, LsKIPK-COM, and LsATPase-COM. Tissues were collected from the bottom of the leaf midrib in the fifth leaf of 3-month-old plants. Tissues were cut into 2-mm^3^ blocks and fixed in 2.5% glutaraldehyde solution for ~24 h. Then, samples were washed with 0.1 M phosphate buffer and fixed with 1% osmium tetroxide. Resin-coated samples were used to make ultrathin sections. Finally, the sections were photographed using a Hitachi H-7650 (https://www.hitachi-hightech.com).

TA-XTPlus (http://www.texturescience.com) was used to determine the maximum shearing force. The leaf midribs were collected from 2-month-old plants in a greenhouse. Four biological replicates were used for each group.

### Cellulose content assay

The cellulose content was investigated using a Cellulose Content Assay Kit (BC4285, Beijing Solarbio Science & Technology). Dry leaves were prepared at 95°C. Samples of ~0.08 g were treated with 80% ethanol and 100% acetone, and the instructions of the kit were followed. Glucose was used as the standard to draw the standard curve. All samples were tested at 620 nm using a spectrophotometer. Finally, the cellulose contents of samples were calculated according to the standard curve.

### GUS activity assay

For the histochemical analysis of GUS activity, we cloned the 1943-bp sequence upstream of the start codon of *LsKIPK*, and the PCR products were recombined into pCAMBIA1301 vector using the ClonExpress II One Step Cloning Kit (Vazyme, Nanjing, China). Primer were listed in Supplementary Data Table S10. The construct was transferred into lettuce as described above. The expression pattern of proLsKIPK::GUS plants was investigated using a GUS staining kit (Coolaber, SL7160), and images of different tissues were captured.

### Data analysis

Each plot point on the graphs represents the value of an individual. Student’s *t*-tests and one-way ANOVA were performed using GraphPad Prism 7.

## Acknowledgements

We thank Peiyao Wu for help in genetic analysis. This work was supported by the National Natural Science Foundation of China award no. 31830079 and the scientific research start-up funding (11020102) from Hubei Hongshan Laboratory.

## Author contributions

S.X. performed the molecular experiments; S.X., B.W., and G.A. performed the genetic analysis; H.K., X.W., and B.W. designed the experiments; X.W. and G.L. helped with data analysis and manuscript writing; S.X. and X.W. wrote the manuscript with help from H.K.

## Data availability

The RNA-seq data have been deposited in the NCBI BioProject database under the PRJNA946888.

## Conflict of interest

The authors declare that they have no competing interests in relation to this work.

## Supplementary data

Supplementary data is available at *Horticulture Research* online.

## Supplementary Material

Web_Material_uhad280
