# Supplementary material for: Lskipk Lsatpase double mutants are necessary and sufficient for the compact plant architecture of butterhead lettuce
Source: Hortic Res. 2023 Dec 28;11(2):uhad280. doi: 10.1093/hr/uhad280 (PMC10873588; doi:10.1093/hr/uhad280)
Supplement: Web_Material_uhad280 [file web_material_uhad280.zip › Supp_ Figures.docx]

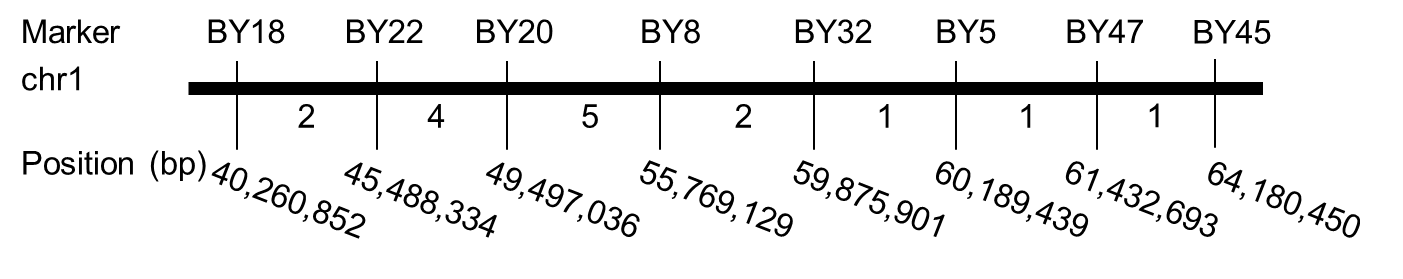


**Figure S1** **Genetic mapping of the gene controlling plant architecture of butterhead lettuce.** Numbers between two neighboring markers refer to the number of recombinants in the F_3_ family.


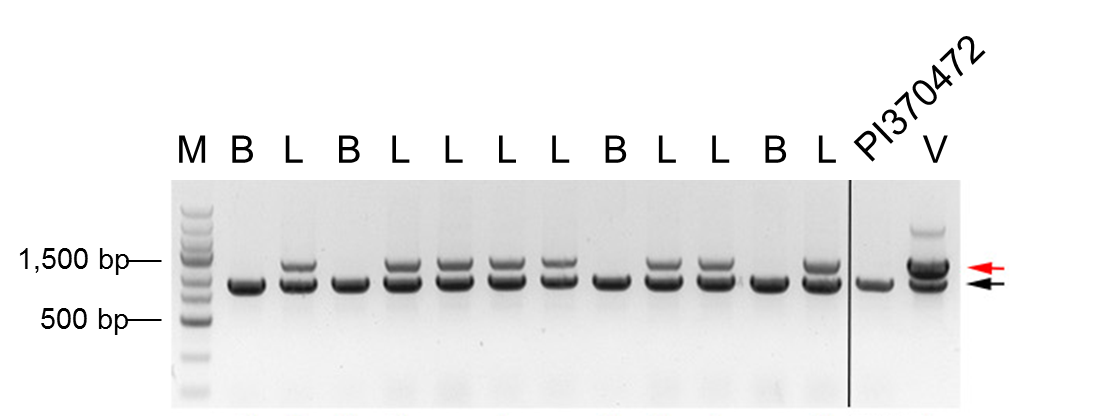


**Figure S2** **Co-segregation assay of transformant of LsKIPK-COM.** “B” refers to the butterhead plant architecture without T-DNA insertion. “L” refers to the looseleaf plant architecture with T-DNA insertion. “V” refers to the complementation vector as positive control. PI370472 refers to butterhead recipient. “M” refers to the DNA marker. The black arrow (903 bp) refers to detection for the DNA quality by using primer pair (LsKIPK-F1/LsKIPK-R1), and the red arrow (1,292 bp) refers to the T-DNA insertion by using the complementation vector primer (LsKIPK-F3/919R).


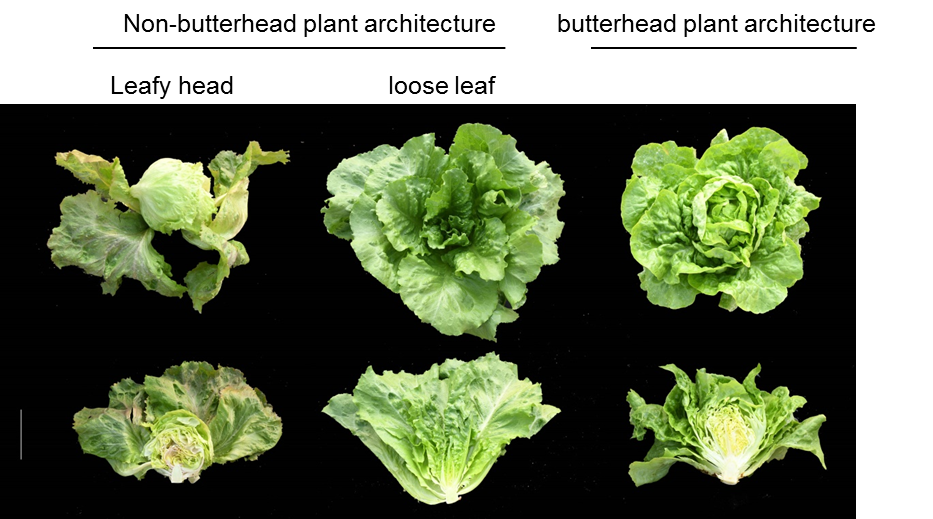


**Figure S3** **The major phenotype of F_2_ population derived from PI577118×PI536734.** Scale bar = 10 cm.


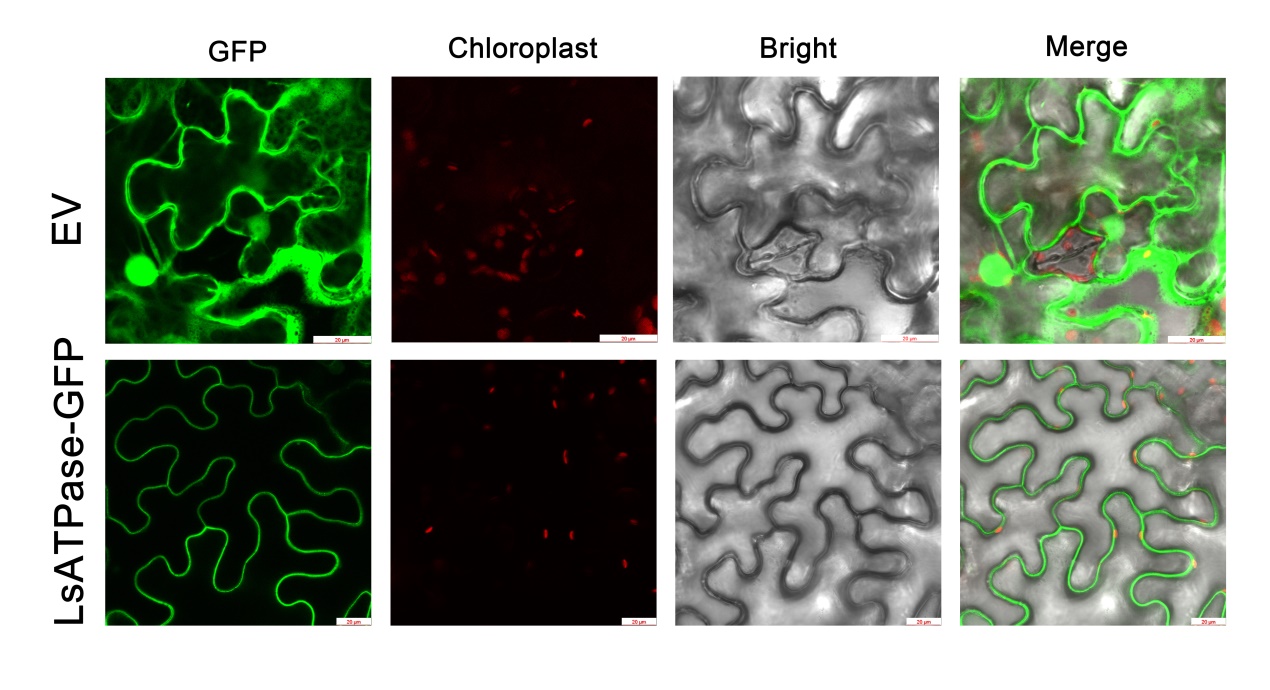


**Figure S4 Subcellular localization of LsATPase**. EV was empty vector of GFP. Scale bar = 20 µm


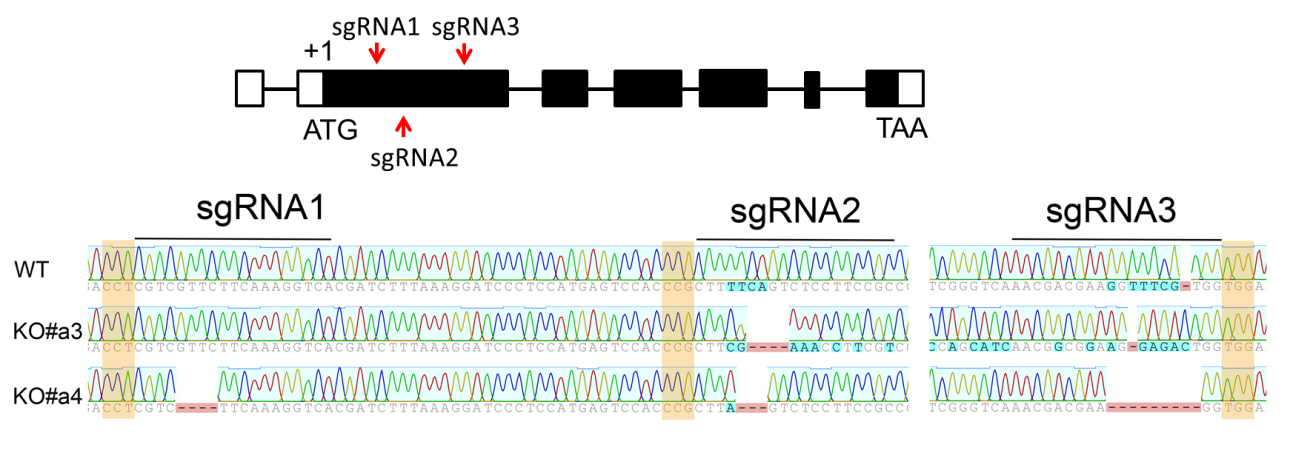


**Figure S5 The sequence of knockout mutant of *Lsatpase*.** Upper panel, the gene structure of *LsATPase* and the position of sgRNAs. Black boxes refer to coding region. Lower panel, Sanger sequencing of two knockout mutants. Dashed lines refer to deletion. Orange shadow refers to the PAM sequences.


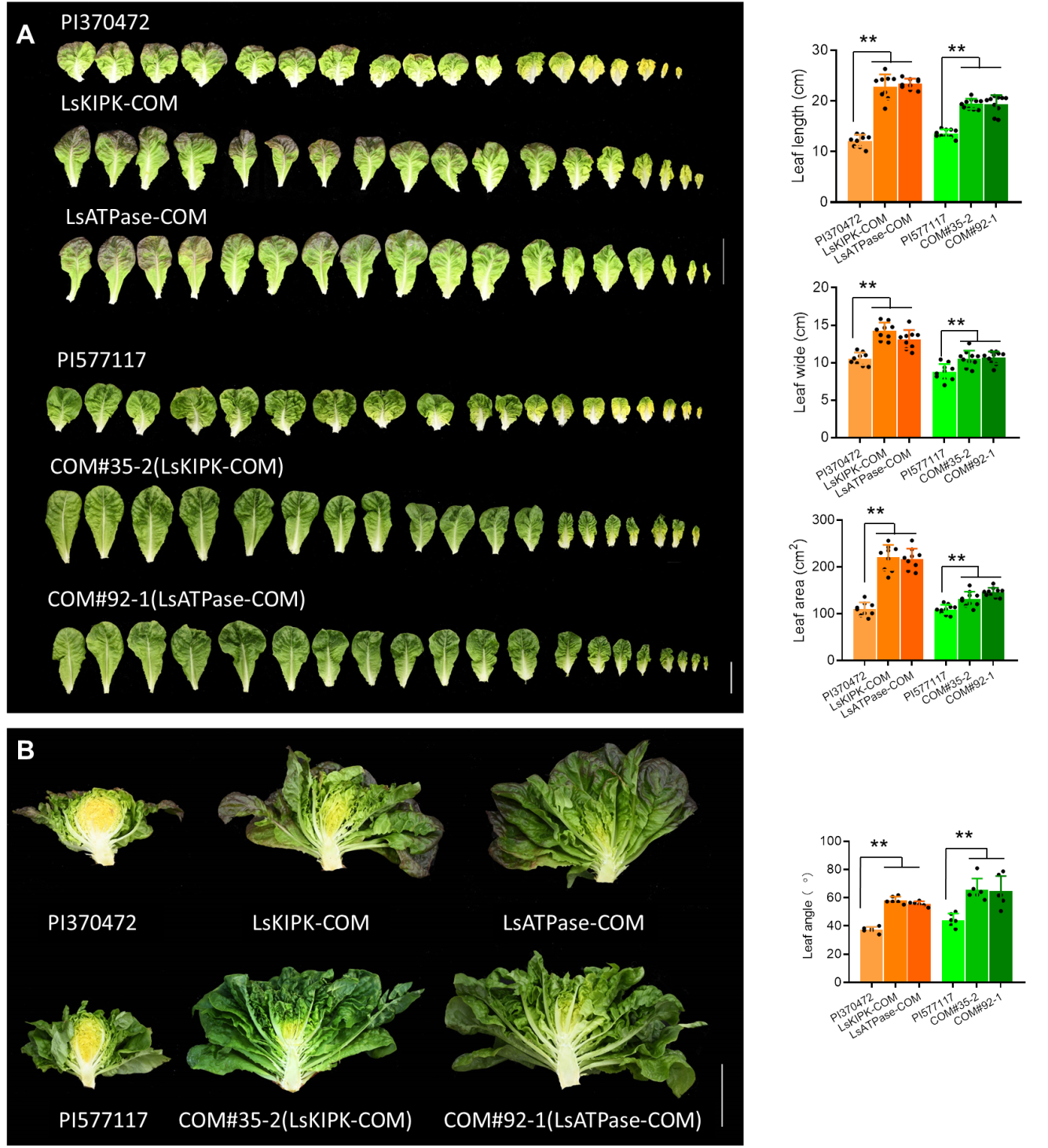


**Figure S6 Morphology of *LsKIPK* and *LsATPase* complementation plants in butterhead lettuce (A)** Leaf morphology of butterhead (PI370472 and PI577117) and complementation plants. Scale bar = 10 cm. (means ± SD; *n* = 9) **(B)** Leaf angle (means ± SD; *n* = 6) of butterhead (PI370472 and PI577117) and complementation plants. Scale bar = 10 cm. ** for *P* < 0.01. Statistical significance was determined by one-way ANOVA.


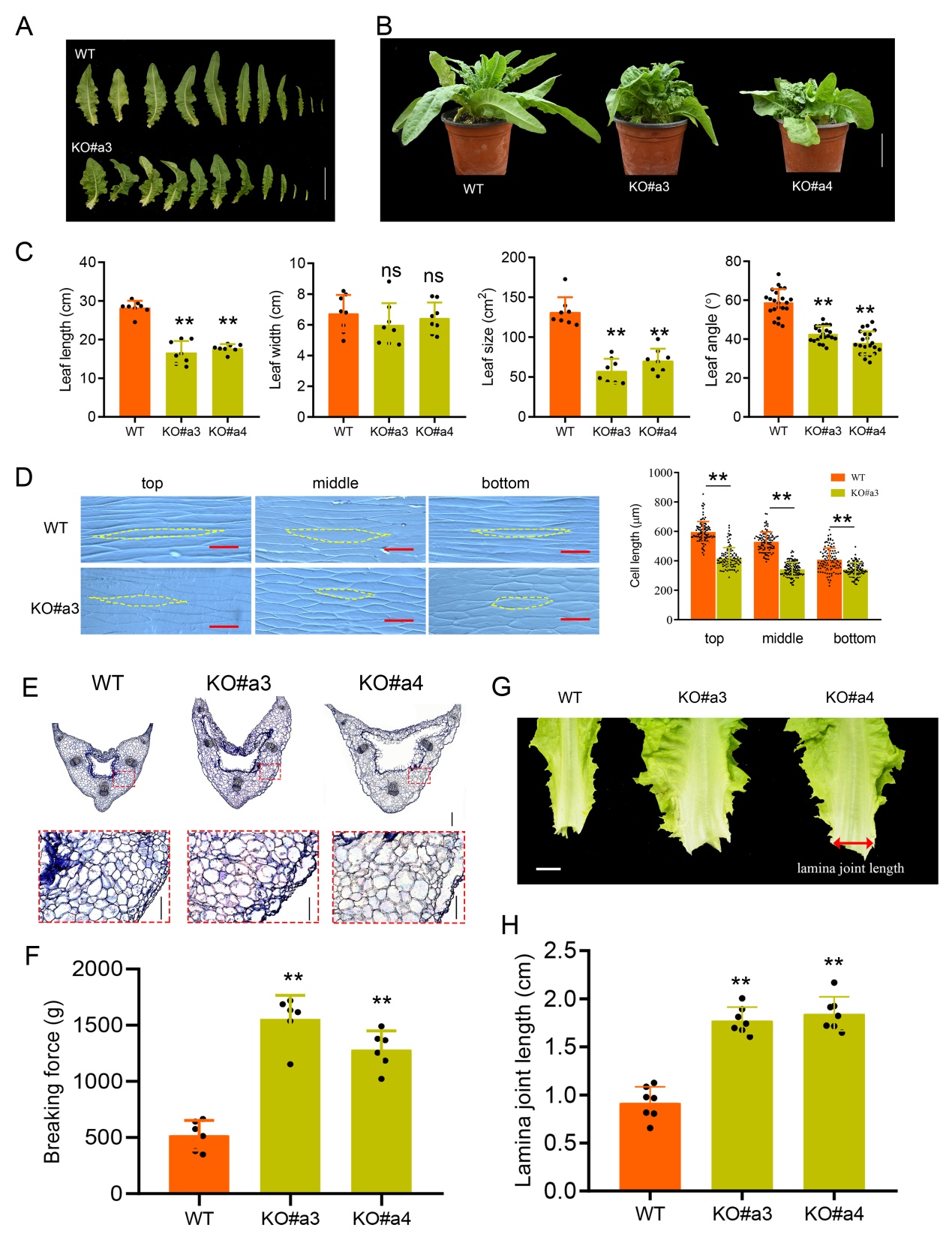


**Figure S7** **Morphology of *Lsatpase* mutant. (A)** Leaf morphology of WT (*Lskipk LsATPase*) and knockout mutant KO#a3 (*Lskipk Lsatpase*). Scale bar = 10 cm. **(B)** Side view of WT and knockout mutant KO#a3 and KO#4 (*Lskipk Lsatpase*). Scale bar = 10 cm. **(C)** Measurement of leaf length (means ± SD; *n* = 8), leaf width (means ± SD; *n* = 8), leaf size (means ± SD; *n* = 8), and leaf angle (means ± SD; *n* = 22) of WT and knockout mutant. **(D)** Epidermal cell length (means ± SD; *n* = 111) of leaf midrib between WT and KO#a3. Scale bar = 200 µm. **(E)** Cross section of leaf midrib for WT and knockout mutant. Scale bar = 400 µm. The rectangle box represents in large view. Scale bar = 200 µm. **(F)** Minimum force (means ± SD; *n* = 4) required to break leaf midrib for WT and knockout mutant. **(G)-(H)** Morphology of lamina joint **(G)** and measurement of lamina joint length (means ± SD; *n* = 7) **(H)** for WT and knockout mutant. Scale bar = 1 cm. * for *P* < 0.05. ** for *P* < 0.01. (d) and (f) Statistical significance was determined by Student’s t test. (c) and (h) Statistical significance was determined by one-way ANOVA.

**
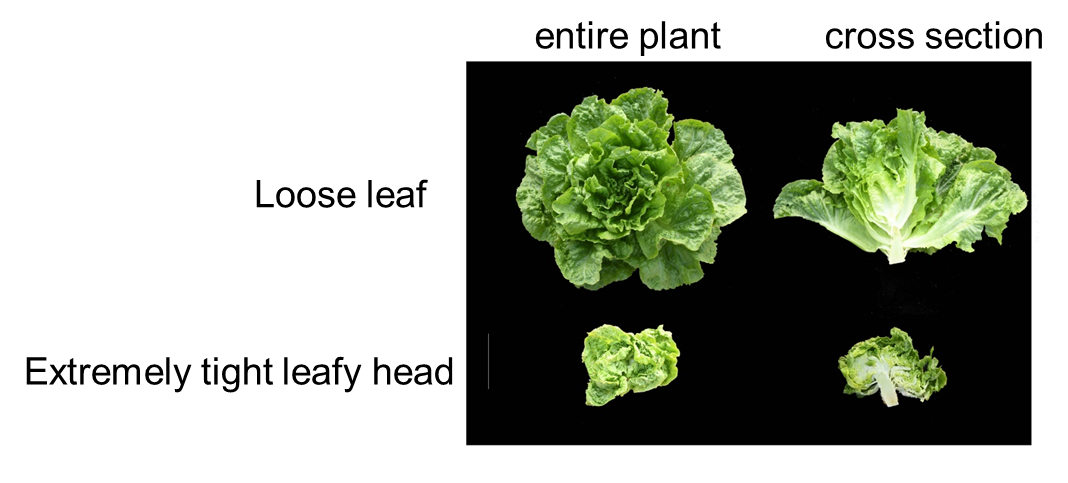
**

**Figure S8 The phenotype of loose leaf individual (*LskipkLskipk*/*LsATPase_*) and extremely tight leaf head individual (*LskipkLskipk*/*LsatpaseLsatpase*) in the F_2_ population derived from PI577118×PI536734.** Scale bar = 10 cm.
